# Transcutaneous Electrical Nerve Stimulation (TENS) Improves the Diabetic Cytopathy (DCP) via Up-Regulation of CGRP and cAMP

**DOI:** 10.1371/journal.pone.0057477

**Published:** 2013-02-28

**Authors:** Liucheng Ding, Tao Song, Chaoran Yi, Yi Huang, Wen Yu, Lin Ling, Yutian Dai, Zhongqing Wei

**Affiliations:** 1 Department of Urology, the Second Affiliated Hospital of Nanjing Medical University, Nanjing, China; 2 Department of Urology, Drum Tower Hospital, Nanjing University Medical School, Nanjing, China; 3 Department of Urology, Chinese PLA 454 Hospital, Nanjing, China; University of Hyderabad, India

## Abstract

The objective of this study was to investigate the effects and mechanism of Transcutaneous Electrical Nerve Stimulation (TENS) on the diabetic cytopathy (DCP) in the diabetic bladder. A total of 45 rats were randomly divided into diabetes mellitus (DM)/TENS group (n = 15), DM group (n = 15) and control group (n = 15). The rats in the DM/TENS and TENS groups were electronically stimulated (stimulating parameters: intensity-31 V, frequency-31 Hz, and duration of stimulation of 15 min) for three weeks. Bladder histology, urodynamics and contractile responses to field stimulation and carbachol were determined. The expression of calcitonin gene-related peptide (CGRP) was analyzed by RT-PCR and Western blotting. The results showed that contractile responses of the DM rats were ameliorated after 3 weeks of TENS. Furthermore, TENS significantly increased bladder wet weight, volume threshold for micturition and reduced PVR, V% and cAMP content of the bladder. The mRNA and protein levels of CGRP in dorsal root ganglion (DRG) in the DM/TENS group were higher than those in the DM group. TENS also significantly up-regulated the cAMP content in the bladder body and base compared with diabetic rats. We conclude that TENS can significantly improve the urine contractility and ameliorate the feeling of bladder fullness in DM rats possibly via up-regulation of cAMP and CGRP in DRG.

## Introduction

As a urinary complication of diabetes, diabetic cytopathy (DCP) is manifested as neurogenic bladder and urethral dysfunction with an incidence of about 50% in diabetic patients [1] ^.^ The exact pathogenesis of DCP is still not clear. Besides myogenic dysfunction of detrusor, lesions of the peripheral nerve of the urinary bladder also play a vital role in the pathogenic mechanisms. Currently, there are no effective treatments for DCP. Conventional drug treatment has noticeable side effects and its curative effect is not sure. Cystostomy has been widely applied in clinical treatment, but it can only improve the clinical symptoms and considerably affect the quality of life [2]. Thus, prevention of the DCP development in early stage is critical for the comprehensive treatment and improvement of the life quality in patients with diabetes. Studies on the safe and effective treatments for DCP are greatly needed.

In 1954, Boyce firstly implanted electrical stimulation into the bladder wall and later in 1963 Caldwell applied electrical stimulation for incontinence treatment [3]. From then on, a series of in vivo electrical stimulation methods have been gradually applied in the treatment of neurogenic bladder [4]. The effects of in vivo electrical stimulation on the regulation of the nerve electrical activity, muscle contractions, nerve excitability and muscle-contraction coupling have been verified. Recently, FDA has approved the sacral nerve stimulation technology for three types of indications: persistent urge incontinence, intractable urgency and urinary frequency syndrome and non-obstructive chronic urinary retention [5]. With continuous technical advancement, electrical stimulation is becoming an important clinical treatment for patients with bladder and urethra dysfunction. However, the traumatic aspect and tolerance of such treatment limited its clinical applications. By far, few studies on the electric nerve stimulation treatment of DCP have been reported. The urodynamics and exact mechanisms of electrical stimulation have not been studied.

In 1980, supra-pubic surface electrical stimulation was firstly applied on interstitial cystitis (IC) to decrease the pain and increase the bladder capacity [6]. In 2004, Yokozuka placed electrical stimulation electrodes on the dorsal S2, S4 sacral foramina surface to treat 18 patients with refractory urinary incontinence, and achieved desired results [7]. However, application of this technology on DCP has been rarely reported and the underlying mechanism is still unknown.

In this study, we first developed a rat model of DCP and investigated the effects of in vivo electrical stimulation on the function of detrusor and the possible mechanisms. The results obtained in this study could provide scientific foundations for electrical stimulation treatment of DCP.

## Materials and Methods

### Animal Models

Animal experiments were performed following the guidelines in the *Guide for the Care and Use of Laboratory Animals* published by the National Institutes of Health (National Institutes of Health publication No. 85–23, revised 1985) and was approved by the Ethics Review Board for Animal Studies of Nanjing Drum Tower Hospital (DTH ERBA 66.01/005A/2010). Sprague-Dawley male rats (n = 45, weight: 180–220 g) were fasted for 18 hours. Diabetes was randomly induced in 30 rats (experimental group) with intraperitoneal injection of STZ (Sigma, St Louis, USA) at a dose of 65 mg/kg. The remaining 15 rats (normal control group) received ice-cold 0.1 mol/L citrate-phosphate buffer (pH 4.2) that was used to dissolve STZ. After 48 hours, 30 rats (experimental group) had a fasting serum glucose concentration of >12 mmol/L, indicating they were diabetic. The rats in the experimental group were further divided into DM/TENS group (n = 15) in which the DM rats were treated with Transcutaneous ENS and DM group (n = 15) in which TENS was not applied. Cystometrogram, histology and contractile analysis were used to assess bladder function. The cAMP concentration of bladder body was quantified as previously described [8].

### Treatment of Animals

On the 10 weeks after the establishment of diabetes, **TENS (**obtained from li he,China) was administered to the rats in DM/TENS group. Electrical stimulation of the urinary bladder was carried out over a period of three weeks. Transcutaneous electrodes were placed suprapubically and below the sacral bone. A pulsed sinusoid current with an intensity of 31 v, frequency of 31 Hz, and duration of stimulation of 15 min was used.

### Detection of CGRP mRNA in Rat Dorsal Root Ganglion by RT-PCR

Total RNA from bladder tissue was isolated by Trizol (Life Technologies, Carlsbad, CA) according to the instructions provided by the manufacturer. cDNA was obtained by using PrimeScripttm RT reagent Kit (TaKaRa, Japan). The mRNA level of CGRP gene was analyzed by quantitative Real-Time RT-PCR using a SYBR® Premix Ex Taq™ System (Takara, Japan). The CGRP primers were 5′-TATATGCAGATGAAAGTCAGGGA (forward) and 5′-ATTAAGCTCACAAGTGACAACATT (reverse). The GAPDH primers (internal control) were 5′-CGATCCCGCTAACATCAAAT (forward) and 5′-GGATGCAGGGATGATGTTCT (reverse).

### Detect ion of CGRP Protein in Rat Bladder Wall and Dorsal Root Ganglion by Western-blot

Tissue and cell was lysed in buffer consisting of 10 mmol/l Tris/HCl (pH 7.2), 150 mmol/l NaCl, 0.1%SDS, 1% (v/v) Triton X-100, 1% sodium deoxycholate, 5 mmol/l EDTA, 1 mmol/l Na_3_VO4, 50 mmol/l NaF, 0.2 mmol/l phenymethylsulfonyl fluoride and protease inhibitor cocktail (complete EDTA-free, Roche). Protein content was determined by BCA protein assay kit (Pierce, MA). Proteins were separated by SDS-polyacrylamide gel electrophoresis (SDS-PAGE) and transferred to polyvinylidene fluoride membranes (Immobilon-P, Millipore, MA). Membranes were blocked in 5% nonfat milk and probed with primary antibodies against CGRP or β-actin for overnight at 4°C followed by 1-hour incubation with HRP–conjugated secondary antibody at 37°C. Bands were visualized by enhanced chemiluminescence (Amersham, NJ) or Chemiluminescence HRP substrate (Millipore, USA) in conjunction with BioMax films (Kodak, USA).

### Statistical Analysis

All values were expressed as mean ± SEM. All data analysis was performed with the use of SPSS 16.0 software. Statistical significance was defined as *P*<0.05 (2-tailed). The distribution of the continuous variables was assessed with the Shapiro-Wilk test. Comparison of parameters between the Ad-synd4 and Ad-null groups was performed by unpaired Student *t* test (when distributions were normal) or Mann-Whitney *U* test (when distributions were not normal). Comparison of parameters among three groups was performed by ANOVA. The authors take full responsibility for the integrity of the data. All authors have read and agreed to the manuscript as written.

## Results

### General Information of the Rats

The bladder weight of the diabetic rats was significantly greater than that of the controls. TENS treatment did not significantly affect the serum glucose levels and body weight during the whole procedure. However, the bladder weight of diabetic rats in the DM/TENS group was significantly lower than that of the DM group (Table 1).

**Table 1:** General information of the rats (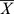
± S).

	*NC* 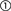	*DM* 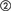	*DM/TENS* 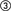
before modeling (Body Weight) (g)	207±9.6	200±9.3	199.6±14
10^th^ week (Body Weight) (g)	393.4±42	181.4±23.6	184.7±25
13^th^ week (Body Weight) (g)	466.9±22	176±40.7	170±35
first week (Blood sugar) (mM)	4.0±0.73	19.2±5.3	19.6±3.9
13^th^ week (Blood sugar) (mM)	4.04±0.46	20.5±4.8	19.67±2.6
bladder wet weight (mg)	175±26	261±56	343±31

In 10^th^ and 13^th^ week: contrast group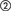 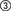 to group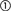, blood sugar is obviously increased and body weight is obviously decreased (p<0.01).contrast group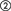 and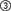, body weight and blood sugar are not statistically different (p>0.05). Contrast group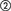 to group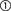, the bladder wet weight is obviously increased, and after the treatment, the bladder wet weight of group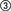 is obviously increased (p<0.01).

### Bladder Capacity and Pressure Measurement

**Figure 1:**
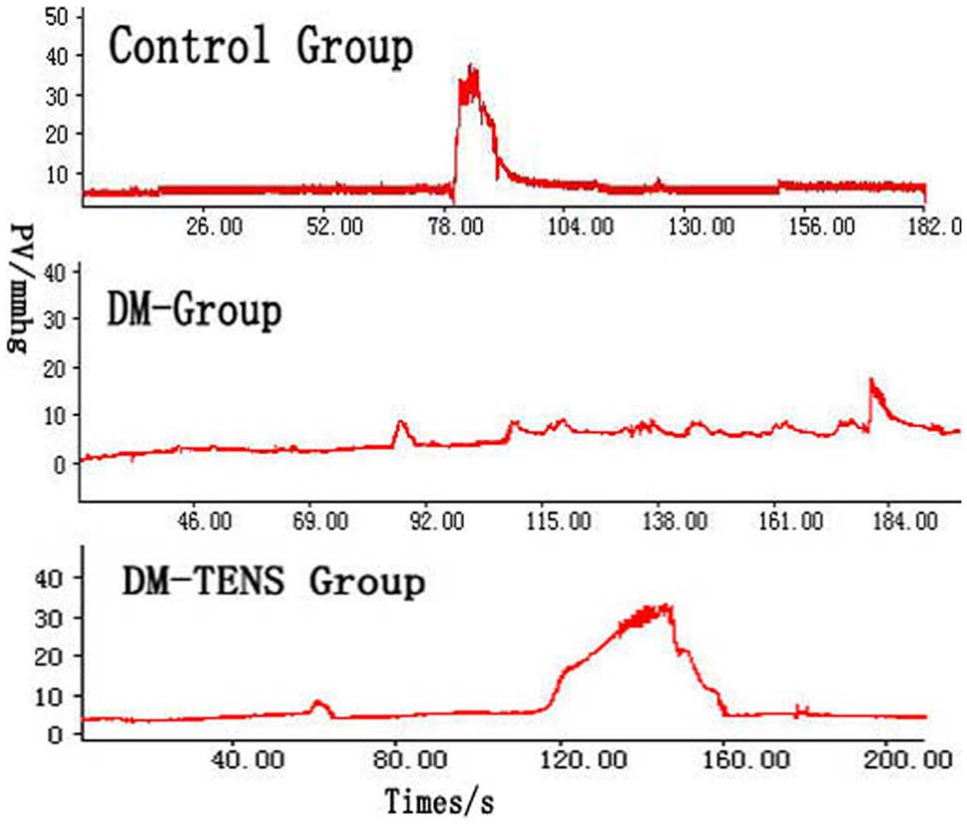
typical urodynamic curves of the rats in each group.

The bladder capacity and R% in DM or DM/TENS group were significantly greater than those in the control group. TENS treatment significantly decreased the volume threshold for micturition, residual urine and V% in the diabetic rats. The maximum intravesical pressure was significantly different between the three groups (Figure 1, Table 2).

**Table 2:** Urodynamic experimental parameters of rats in each group (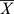 ± S).

Group	N	MCC(ml)	RV (ml)	R%	Maximum Pdet(mmHg)
NC group	10	0.59±0.06	0.14±0.044	24.2±8.7	43.2±7.0
DM group	10	1.4±0.40 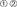	0.58±0.16 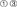	42.8±8.7 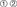	19.5±6.5 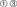
DM/TENS group	10	0.93±0.26 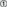	0.30±0.10 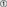	32.8±6.7 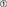	30.0±3.8 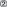

DM group and Control group: 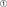 p<0.01; DM group and DM/TENS group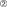 p<0.05; 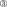 p<0.01.

DM group and DM/TENS group: 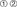 p<0.05; 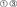 p<0.01. DM/TENSDM/TENS group and Control group: 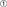 p<0.05; 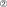
p<0.01.

### Contractile Responses of Detrusor Strips to EFS and ACh

**Figure 2:**
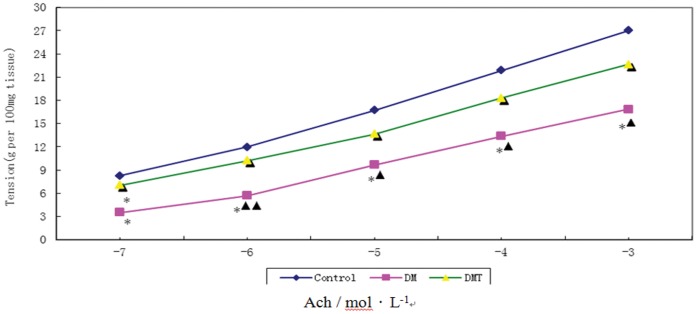
Concentration-response relationships for bladder body strips from control, diabetic-treated rats. (*P<0.01 VS control group, ^▴▴^ *P*<0.01 VS DM/TENS group, ^▴^ *P*<0.05 VS DM/TENS group).

**Figure 3:**
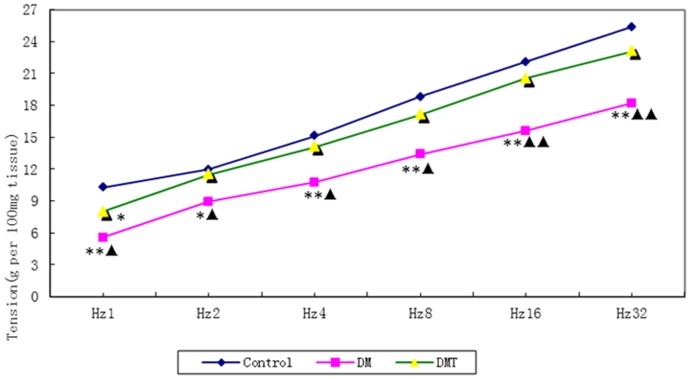
Frequency-tension curve of electrical field stimulation (**P<0.01 VS control group, *P<0.05 VS control group, ^▴▴^ ***P*** **<0.01 VS DM/TENS group,** ^▴^ ***P*** **<0.05 VS DM/TENS group).**

The peak force in response to ACh increased in the bladder strips as the concentration of ACh increased. The bladder strips from the diabetic rats produced more force than the same size strips from the controls. The sigmoidal concentration-response curve was significantly shifted upward in the bladder strips of the DM group. TENS reduced the contractile force mildly in the diabetic bladder strips (Figure 2). The peak force in response to EFS increased in the bladder strips as the frequency of stimulation increased. Although the bladder strips from the diabetic rats produced more force in absolute terms than the same size strips from the control rats, the diabetic bladder strips (per 100 mg) actually produced less force at the different frequencies in terms of each strip weight than the control strips. The frequency-response curve was significantly shifted downward in the bladder strips of DM group. TENS significantly potentiated the neurogenic contraction in the diabetic bladder (Figure 3).

### TENS Up-regulates cAMP Content of the Bladder

**Figure 4:**
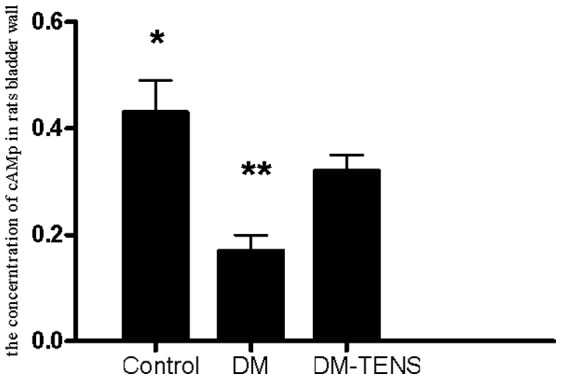
Quantification of cAMP concentration of the bladder in rats DRG of each group (**P<0.01 VS DM/TENS group, *P<0.05 VS DM/TENS group).

The concentration of cAMP in the base and body of the diabetic bladder was significantly lower than that of the controls. TENS significantly up-regulated the cAMP content in the bladder body and base compared with diabetic rats, although cAMP content in the DM/TENS groups was still lower than that in the controls (Figure 4).

### TENS Up-regulates the Expression of CGRP in the Dorsal Root Ganglion

**Figure 5:**
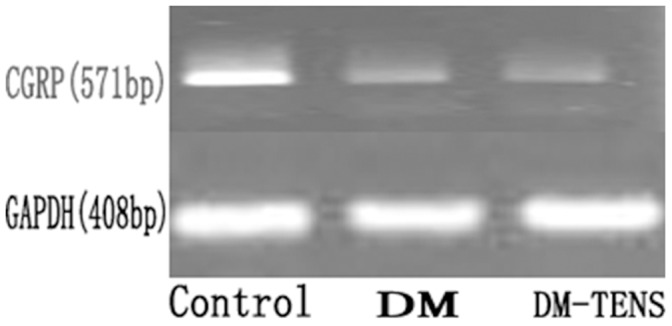
RT-PCR of CGRP in rats DRG of each group (**P<0.01 VS DM/TENS group, *P<0.05 VS DM/TENS group).

**Figure 6:**
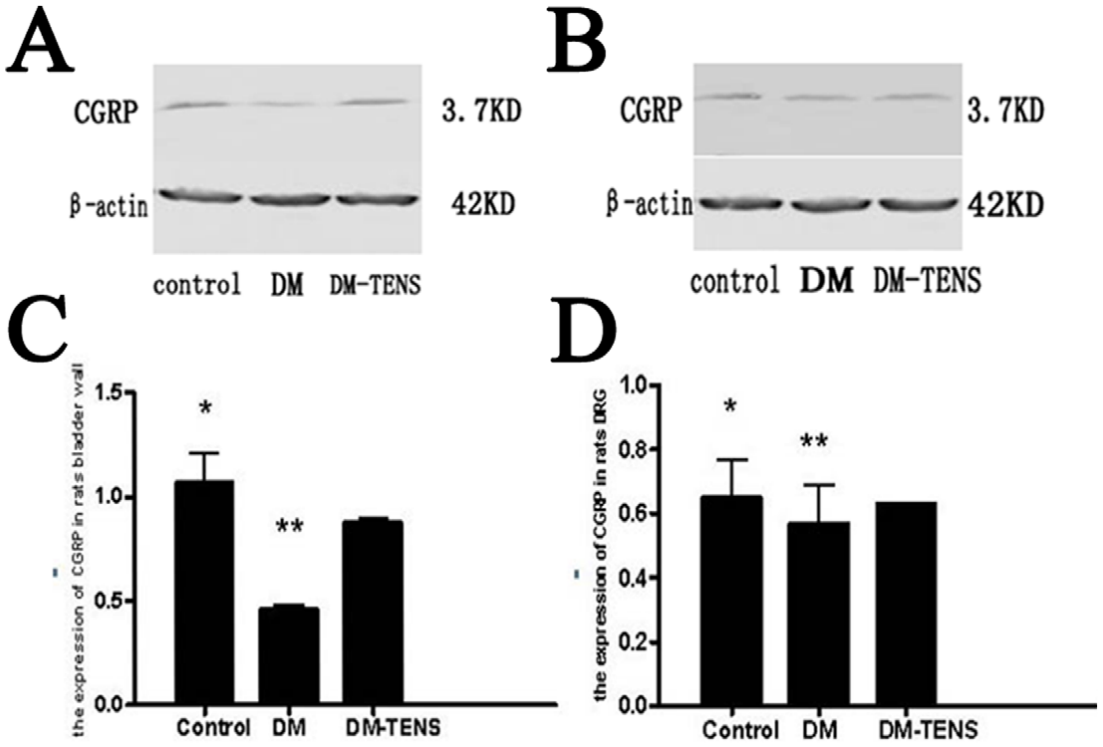
Quantification of CGRP protein level in rats bladder wall (A&C)and DRG(B&D) in control, DM and DM/TENS group, (**P<0.01 VS DM/TENS group, *P<0.05 VS DM/TENS group).

The CGRP mRNA level in DRG of DM group was significantly lower than that in the control group. The CGRP mRNA level in the DM/TENS group was significantly higher than that of DM group. However, CGRP mRNA level in the DM/TENS group was still significantly lower than that in the control group (Figure 5, Table 3). Similar results were obtained for the CGRP protein level (Figure 6).

**Table 3:** mRNA content of CGRP in rats DRG of each group.

Group	CGRP/β-action(bladder wall)
NC	1.13±0.11*)
DM	0.54±0.02
DM/TENS	0.79±0.03*) **)

^*)^DM/TENS group and DM group P<0.01.

^**)^DM/TENS group and Control group P<0.05.

## Discussion

DCP is a common complication of diabetes with an incidence of 40%–100% in diabetic patients. The incidence of DCP is also as high as 25% in patients with stable blood sugar [9]. Previous studies have demonstrated that the dysfunction of diabetic bladder is mainly caused by the bladder detrusor dysfunction and peripheral autonomic neuropathy. The key pathogenic aspects of DCP are the decline of excitability, compliance, and systol of detrusor cells as a result of the synergistic effect of the myogenic factors and neurogenic factors [10].

Clinical and experimental studies on the treatment of DCP have been mainly focusing on the acceleration of nerve regeneration and delay of the detrusor atrophy. The main treatment for DCP is pharmacotherapy including blood glucose control, nerve nourishment, promotion of bladder contraction and circulation and application of antibiotics. Although the treatment (surgical and conservative) of diabetic neurogenic dysfunctions of micturition can protect upper urinary tract function, the DCP could be recurrent.

During the past decades, a number of studies have investigated the therapeutic efficacy of neuromodulation (e.g., stimulation of pudendal nerve, sacral nerve, transcutaneous and intravesical electrical stimulation) of the bladder and established an artificial somatic-visceral reflex pathway as a relatively effective means for neurogenic bladder [11]–[15]. It appears that modulation of spinal cord reexes and brain networks is involved in the neuromodulation [16], but the exact mechanism remains to be elucidated Neuromodulation predominantly involves the areas associated with sensorimotor learning, which might become progressively less active during the course of chronic neuromodulation. Moreover, the sympathetic nervous system might play a role as low-frequency pudendal nerve stimulation occurs in cats with chronic spinal cord injury. It has been well demonstrated that sensory stimulation of the abdominal skin by pinching inhibits gastric (bladder) motility via increasing sympathetic efferent activity in rats [17], [18]. Some studies also reported that intravesical electrical stimulation can ameliorate children’s bladder contractile function [^19^] **,** and one previous study showed that electrical stimulation of the stomach could contribute to improving the gastroparesis in patients with diabetes [20]. However, non-invasive electrical stimulation has not been applied to improve the voiding dysfunction in rats with DCP. Therefore, in this study, we applied non-invasive electrical stimulation in the surface to improve the voiding dysfunction in rats with DCP, and explored the underlying mechanisms.

We found that the increase of bladder weight was correlated with an increase of the bladder threshold capacity and the residual urine. The weight and detrusor contractility were decreased as early as 10 weeks in the rat DM model. However, after 3 weeks’ treatment with TENS, the bladder threshold capacity and residual urine were decreased significantly, suggesting that electrical stimulation therapy could improve the bladder dysfunction caused by DM.

cAMP is an important intracellular second messenger of β-adrenergic nerve and several NANC nerve pathways. During urine storage, cAMP plays an important role in the regulation of the diastolic function of detrusor and the maintenance of the filling sensation and compliance of bladder. Meanwhile, cAMP also has the ability to relax bladder neck and decrease the bladder outlet resistance in voiding [21]. Therefore, the interaction of transmitter and receptor of independent nerve in lower urinary tract could be damaged by the reduced concentration and activity of cAMP. Our in vivo results demonstrated that cAMP level of bladder decreased significantly in the rat DM model, meanwhile cAMP level is consistent with the detrusor systolic and diastolic dysfunction and the change of the compliance of bladder.

CGRP is an NANC substance and belongs to the member of the calcitonin gene-related peptide family. CGRP is an important neurotransmitter of sensory nerves involved in the pain signal transduction, and it is widely distributed in the spinal dorsal horn and primary afferent fibers associated with the surface sense. In the dorsal root ganglion, CGPR is present in 40–50% of the neurons. CGRP also plays a role in the the proliferation of Schwann cells [22]–[24]. Contractile response of bladder muscle strips to electrical stimulation was reduced in 4^th^, 8^th^ and 12^th^ week of STZ administration. The reduction was most obvious in the 12^th^ week, suggesting that it may be resulted from the decline of the release of neurotransmitter in non-cholinergic motor neurons [25]. Immunohistochemical analysis showed that CGRP levels were significantly reduced in the bladder wall of diabetic rats, especially in the submucosal plexus (Yu Gong et al., 2000), suggesting that the peptidergic neurotransmitters in the bladder wall of diabetic rats are obviously abnormal. The abnormal peptidergic neurotransmitters may impair the sensory function of bladder, leading to the development of DCP.

Our in vivo and in vitro studies showed that expression of CGRP in bladder wall and DRG was significantly reduced in the DM group compared to control group. However, CGRP expression in the DM group was enhanced by three weeks of in vitro electrical stimulation therapy. These results indicate that CGRP, as a sensory nerve neurotransmitter, plays a critical role in the pathological process of urination dysfunction in diabetic rats. We have also observed that the expression of CGRP in bladder wall and DRG of rats in each group was increased or decreased simultaneously with synchronization characteristics. Thus, obvious obstacle may not exist in the process of transportation and release of CGRP in axon. Therefore, we speculate that effective transport and release process of CGRP protein may be protected by the in vitro electrical stimulation, which could promote the repair of impaired peripheral nerve fibers.

The contractility of muscle strips in DM/TENS group and control group was significantly stronger than that in DM group, which may be related with the enhancement of the conduction velocity of nerve fibers, the improvement of Ca^2+^-ATPase and the adaptive changes of Ca^2+^ release-uptake kinetics [26]. Prior studies demonstrated that the detrusor strips from DM rats which were stimulated by ACH had stronger contractility than that from the control group [8], which is not consistent with our present study. It may be related with difference in the tolerance and duration of diabetes. Our experiments were conducted after the successful modeling of an average of 10 weeks, therefore the complications of diabetes appeared to be more typical [1]. Because the change in the course of the DCP is time-dependent, the amount and activity of acetylcholine receptors in the detrusor strips from DM group are further decreased without the compensatory increases. Therefore, we speculated that it may be resulted from the increased activity of cholinergic receptors, the promoted release of Ach and the decreased expression of Ach [^27^].

In summary, our results indicate that DCP could be improved by in vitro electrical stimulation. The underlying mechanism for the DCP improvement may be due to (1) increases of cAMP in bladder, which could modulate the signaling pathways of neurotransmitter and receptors and (2) increases of CGRP expression in bladder wall and DRG, leading to the enhancement of the contractility of the detrusor and sense of bladder filling.
